# Infecting mosquitoes alters DENV-2 characteristics and enhances hemorrhage-induction potential in *Stat1*^*-/-*^ mice

**DOI:** 10.1371/journal.pntd.0009728

**Published:** 2021-08-27

**Authors:** Ka Wan Cheang, Wen-Yu Chen, Betty A. Wu-Hsieh, Shin-Hong Shiao

**Affiliations:** 1 Graduate Institute of Immunology, National Taiwan University College of Medicine, Taipei, Taiwan; 2 Department of Tropical Medicine and Parasitology, National Taiwan University College of Medicine, Taipei, Taiwan; Baylor College of Medicine, UNITED STATES

## Abstract

Dengue is one of the most prevalent arthropod-borne viral diseases in humans. There is still no effective vaccine or treatment to date. Previous studies showed that mosquito-derived factors present in saliva or salivary gland extract (SGE) contribute to the pathogenesis of dengue. In this study, we aimed to investigate the interplay between mosquito vector and DENV and to address the question of whether the mosquito vector alters the virus that leads to consequential disease manifestations in the mammalian host. DENV2 cultured in C6/36 cell line (culture-DENV2) was injected to *Aedes aegypti* intrathoracically. Saliva was collected from infected mosquitoes 7 days later. Exploiting the sensitivity of *Stat1*^*-/-*^ mice to low dose of DENV2 delivered intradermally, we showed that DENV2 collected in infected mosquito saliva (msq-DENV2) induced more severe hemorrhage in mice than their culture counterpart. Msq-DENV2 was characterized by smaller particle size, larger plaque size and more rapid growth in mosquito as well as mammalian cell lines compared to culture-DENV2. In addition, msq-DENV2 was more efficient than culture-DENV2 in inducing *Tnf* mRNA production by mouse macrophage. Together, our results point to the possibility that the mosquito vector provides an environment that alters DENV2 by changing its growth characteristics as well as its potential to cause disease.

## Author summary

The transmission cycle of dengue virus involves a complex interaction of the virus with the mosquito vector and the human host. DENV enters the mosquito through an infectious blood meal. The virus first infects and replicates in the mosquito midgut before it is translocated to the hemolymph and ultimately reaches the salivary gland. The virus is then released to the saliva and transmitted to the human host through a mosquito blood meal. Previous studies showed that mosquito saliva, salivary gland proteins or mosquito bite modulates arbovirus infection in the mammalian host. Here, we investigate the interplay between the mosquito vector and DENV and address whether the virus is altered in the mosquito vector that consequentially influences disease manifestations in the mammalian host. By excluding the effect of saliva, we demonstrated that after infecting *Aedes aegypti*, DENV2 acquired the potential to induce more severe hemorrhage in *Stat1*^*-/-*^ mice. Msq-DENV2 exhibited smaller particle size, larger plaque size and more rapid growth rate, and was also more efficient in inducing macrophage *Tnf* production compared to culture-DENV2. Taken together, our data support the notion that mosquito *A*. *aegypti* alters DENV2 by changing its growth characteristics as well as its potential to cause disease.

## Introduction

Dengue is a mosquito-borne viral disease that affects humans in both the tropics and subtropics. It is estimated that there are over 390 million dengue virus (DENV) infections per year globally [1] and 500,000 of the infected require hospitalization [2–4]. The World Health Organization classifies dengue into dengue and severe dengue. Approximately one-third of dengue patients may have hemorrhage manifestations, the spectrum includes mild skin hemorrhage, gingival or nasal bleeding, gastroinetestinal bleeding and severe hemorrhage [5]. Patients with severe dengue experience plasma leakage, severe bleeding, fluid accumulation, respiratory stress or organ impairment and are at risk of death [5]. It is, therefore, evident that hemorrhage is a clinically significant sign of DENV infection.

The *Aedes* mosquitoes, principally *Aedes aegypti* (*A*. *aegypti*) and *A*. *albopictus*, are the primary vectors that transmit DENV to humans [4]. DENV is taken up by the mosquito during an infectious blood meal. The virus infects the midgut epithelium and replicates before it traverses the basal lamina into the hemolymph and disseminates throughout the mosquito body. It reaches the salivary glands from where it is released to the saliva and transmitted to the mammalian host [6]. Of the interplay between mosquitoes, virus, and mammalian host, many studies focused on the impact of mosquito saliva, salivary gland proteins or mosquito bites on the establishment of infection or disease progression in the mammalian host. Cox et al. compared infection of humanized IRF3^-/-^7^-/-^ mice with DENV through infected mosquito bite and by needle injection. They found that mice infected by mosquito probing exhibited more severe disease, higher and more sustained viremia, erythema and thrombocytopenia compared to infection through needle inoculation [7]. A different study also showed that infecting IRF3^-/-^7^-/-^ mice with DENV through infected mosquito bite resulted in higher viremia titer two days preceding the peak [8]. Probing by naïve mosquitoes before needle injection of DENV also increased viremia titer but downregulated TLR7, RelA, IFN-γ and IL-10 transcripts during the first 3 to 6 h of infection compared to needle delivery of the virus [9]. These studies demonstrated that mosquito salivary factors delivered through probing modulate DENV replication, immune response as well as disease progression in the host. However, how the mosquito vector impacts the pathogenicity of the virus has not been addressed. Here we aimed to focus our study on the interplay between the mosquito vector and DENV and to address the question of whether the mosquito vector alters the virus so that it affects the consequential disease manifestations in the mammalian host.

A number of mouse models were established to study dengue. We established a dengue hemorrhage mouse model in immune competent mice by injecting DENV intradermally [10]. Hemorrhage developed in 3 days after infection. Skin cryosections showed that hemorrhage was accompanied by macrophage production of TNF and the presence of apoptotic endothelial cells. TNF deficiency or depletion by neutralizing antibody diminished DENV-induced hemorrhage [10]. Immunodeficient mice including AG129 mice that lack type I and II IFN receptors, *Ifnar*^*-/-*^ mice lacking type I IFN receptor, IRF3^-/-^7^-/-^ and *Stat1*^*-/-*^ (signal transducers and activators of transcription 1) have been demonstrated to be susceptible to DENV infection [8,11–14]. DENV-induced viremia, hemorrhage, thrombocytopenia, viral dissemination, and death were observed in these deficient mice [11–14]. In this study, by employing *Stat1*^*-/-*^ mice and applying intradermal route of inoculation, we investigated whether infecting mosquito alters the hemorrhage-induction potential of DENV in the mammalian host.

We have previously devised a unique method to collect saliva from large numbers of either infectious or uninfected mosquitoes [15]. Infectious saliva was collected from *A. aegypti* mosquitoes that were previously injected with DENV intrathoracically. Comparing DENV from infectious saliva (msq-DENV) to culture grown DENV (culture-DENV) of their particle size, plaque size and growth characteristics, we found that infecting mosquitoes altered the characteristics of the virus. Moreover, msq-DENV was more efficient than culture-DENV in inducing *Tnf* in mouse macrophages and induced more severe hemorrhage in *Stat1*^*-/-*^ mice. Our results demonstrate that *A*. *aegypti* alters the characteristics of DENV and enhance its potential in causing disease in the mammalian host.

## Materials and methods

### Ethics statement

*Stat1*^-/-^ mice on C57BL/6 background (from Dr. C.-K. Lee, Graduate Institute of Immunology, National Taiwan University College of Medicine, Taipei, Taiwan) were bred in the Laboratory of Animal Center at National Taiwan University College of Medicine (Taipei, Taiwan) in specific pathogen-free (SPF) environment. Mouse study was carried out in strict accordance with the recommendations in the Guidebook for the Care and Use of Laboratory Animals, The Third Edition, 2007, published by The Chinese-Taipei Society of Laboratory Animal Sciences. All animal procedures and experimental protocols were approved by AAALAC-accredited facility, the Committee on the Ethics of Animal Experiments of the National Taiwan University College of Medicine (IACUC approval No: 20140275).

### Virus

DENV serotype 2 strain 16681 (DENV2-16681) used in the study was originally isolated from a Thai patient who suffered from DHF [16]. The virus was passaged in mosquito C6/36 cell line as previously described [10]. In brief, C6/36 cells in 2.5 × 10^6^ were seeded in 10-cm tissue-culture dish two days before infection by DENV at MOI of 0.01. Five days later, culture supernatant was collected and concentrated with Amicon Ultra Centrifugal Filter (10,000 MWCO; Millipore) by centrifugation at 1,000 g at 4°C for 30 min. Viral titer was determined by plaque assay using BHK-21 cell line [4].

### Mosquitoes

The *Aedes aegypti* UGAL/Rockefeller strain was used in this study. The mosquitoes were raised as previously described [17,18]. Briefly, mosquitoes were provided with 10% sucrose solution and maintained at 28°C in 60–70% humidity with a 12/12 h light/dark cycle. Males and females were kept in the same cage until given a blood meal. Female mosquitoes at 3–5 days post-eclosion were allowed to feed on anesthetized ICR mice (Institute of Cancer Research, USA) to initiate egg development. The ICR mice used in this study were obtained from the Laboratory of Animal Center at National Taiwan University College of Medicine (Taipei, Taiwan).

### Infection of mosquitoes with DENV

Female mosquitoes at 3–5 days post-emergence were anesthetized by CO_2_. Each mosquito was intrathoracically inoculated with 69 nl of DENV-2 16681 (6.9 × 10^2^ PFU in 69 nl) grown in C6/36 cell culture using a Nanoject II injector (Drummond, Broomall, Pennsylvania, USA) as described previously [19]. Mosquitoes were then transferred into cylindrical containers fitted with nylon mesh on the top. Saliva was collected from infected mosquitoes on day 7 after infection. Viral titer was determined by plaque assay [19].

### Collection of mosquito saliva

Collection of mosquito saliva was performed as previously described [15]. Approximately total of 4,000 DENV2-infected mosquitoes were used for infectious saliva collection. Intrathoracically infected as well as naïve mosquitoes (approximately 200 each per experiment) were starved for 12 h before saliva collection. On the day of saliva collection, mosquito meal was prepared in 1000 μl RPMI medium (with no phenol red) containing 10 μl of adenosine triphosphate (as phagostimulant) at 2 mM (final concentration of 20 μM). Meal wrapped in Parafilm-M membrane was warmed to 37°C on a dry heating plate before being offered to mosquitoes. Infected and naïve mosquitoes were fed on separate meals through Parafilm-M membrane for 1 h. The meals which now contained naïve mosquito saliva or infectious saliva if the mosquitoes were infected were placed in microtubes and centrifuged at 8,000 g at 4°C for 3 min. The empty Parafilm-M membrane was removed and the saliva-containing meal (approximately 200 μl) was collected, concentrated 4-5-fold and sterilized by passage through a 0.22 μm GV Durapore Centrifugal Filter Units (Millipore). The meal was dispensed into Eppendorf tubes in aliquots and stored at −80°C until use.

Infectious saliva at 180 μl was dropped on a Parafilm-M membrane that was placed on a 6-cm tissue-culture dish. The infectious saliva was then exposed to ultraviolet (UV) light in a laminar flow hood for 30 min [10]. After exposure, saliva was centrifuged at 8,000 g at 4°C for 3 min and filter-sterilized through 0.22 μm GV Durapore. The UV-inactivated infectious saliva was dispensed into Eppendorf tubes in aliquots and stored at −80°C until use. The effect of UV inactivation on virus infectivity was confirmed by plaque assay.

### Dengue hemorrhage mouse model

*Stat1*^*-/-*^ mice were shaved and intradermally inoculated at four sites on the upper dorsal skin with 6 × 10^3^ PFU in 400 μl of DENV2 that was propagated in C6/36 cells (culture-DENV2) alone, culture-DENV2 premixed with naïve saliva (4 μg saliva protein), saliva obtained from DENV-infected mosquitoes, or culture-DENV2 pre-mixed with UV-inactivated infectious saliva. Hemorrhage manifestations in the dorsal skin, subcutaneous tissue and abdominal skin were observed on day 6 after infection. The hemorrhaged areas were quantified by ImageJ software as described previously [19]. The percentage of hemorrhaged areas = hemorrhaged areas/total area of the selected region (S3 Fig).

### Hematoxylin-eosin (H&E) staining

Dorsal and abdominal skins from hemorrhaged mice were cut into 1cm x 1cm pieces, fixed in 4% neutral formalin and left in room temperature for 24 h. The skins were then embedded in paraffin, sectioned at 4 μm thickness and mounted on glass microscope slides. The sections were stained with hematoxylin and eosin and read by the Laboratory of Animal Center at National Taiwan University College of Medicine (Taipei, Taiwan).

### Cultivating msq-DENV2 in cell culture

C6/36 cells at 1 × 10^7^ were seeded in 75T tissue-culture flasks. After grown to full confluence, C6/36 cells were infected by infectious saliva (msq-DENV2) at MOI of 0.01. Seven days later, culture supernatant (msq-DENV2-P1) was collected and filter-sterilized through 0.22 μm GV Durapore Syringe Filter Units. Viral titer was determined by plaque assay using BHK-21 cell line.

### Transmission Electronic microscopy

Msq-DENV2-P1 at 10^7^ PFU was fixed with the same volume of 4% paraformaldehyde overnight. The next day, the viral samples were 10-fold-concentrated with Vivaspin 500 (3 kDa MWCO; GE Healthcare) by centrifugation at 13,500 g at 4°C for 30 min. To negative stain, 10 μl of the concentrated viral sample was aspirated onto mesh nickel grids (FCF300-Ni, Electron Microscopy Sciences), followed by staining with 40 μl of 2% uranyl acetate for 30 sec. Images were viewed with a Hitachi H-7650 transmission electron microscope (TEM) using an accelerating voltage of 120 kV. The size of viral particles was quantified by ImageJ software.

### Plaque assay and plaque size measurement

Plaque assay was conducted on BHK-21 cells [19]. BHK-21 cells at 6 × 10^4^ were seeded in the wells of 24-well tissue-culture plate and let sit in incubator overnight. The cell monolayers were rinsed with PBS. Ten-fold serially diluted culture-DENV2, infectious saliva, UV-inactivated infectious saliva, or msq-DENV2-P1 at 200 μl was added to the BHK-21 cell monolayers for absorption for 2 h. After absorption, 800 μl of overlay agarose (2% sterile UltraPure LMP Agarose and 2% DMEM, 1:1) was added and allowed to gel at room temperature for 20 min before the plates were returned to the incubator. After incubation for 5 days, the plates were fixed with 4% paraformaldehyde and left at room temperature for 2 h. Agarose overlays were then removed and the plates were stained with 1% crystal violet for 1 min. The number of plaques were quantified by manual counting. To measure plaque size, the plates were scanned using an image scanner. The wells that had 12–15 plaques were selected for plaque size measurement. The total area of the entire well and the area of the plaques were quantified by ImageJ software (S3B Fig). Plaque size = (pixel number of a plaque/pixel number of the whole well) × 1.9 cm^2^ (area of the whole well).

### DENV2 growth in mammalian and mosquito cells

C6/36 cells, ATC10 cells or BHK-21 cells at 1 × 10^6^ were seeded in 24-well culture plates one day before infection. After grown to full confluence, cell monolayers were infected with culture-DENV2 or msq-DENV2-P1 at a MOI of 0.1 (C6/36 and ATC10) or MOI of 0.01 (BHK-21) for 2 h. The inoculum was removed, and the monolayer was rinsed with PBS prior to the addition of complete medium. Culture supernatant was then collected at 6, 24, 48, 72 h after infection. Samples were stored at -80°C until determination of titer by plaque assay.

### Western blot analysis and Coomassie blue staining

ATC10 cells were infected with culture-DENV2 or msq-DENV2-P1 at a MOI of 0.1. Culture supernatant was collected at different time points. To detect E protein secreted by the viruses in culture, 20 μl of culture supernatant was loaded and subjected to electrophoresis at 10% SDS-polyacrylamide gel and the proteins were transferred to 0.22 μm PVDF membrane (Millipore) [12]. Membrane was blocked with 5% milk in PBS-T (1 × PBS, 0.2% Tween20) at room temperature for 30 min and incubated in PBST buffer containing mouse anti-E mAbs (1:10,000 dilution, GTX127277, GeneTex) at 4°C overnight. Membrane was then incubated in PBST buffer containing horseradish peroxidase-conjugated anti-mouse IgG (1:5000 dilution) for 1 h. Western Chemiluminescent HRP (Advansta Inc.) was used as substrate and protein signals were detected by X-ray film (Fujifilm). The intensity of the protein blot was quantified by ImageJ software.

To compare the total protein loaded in each lane, the membrane was washed after electrophoretic separation in PBST three times before stained with Coomassie blue (0.1% Coomassie Brilliant Blue R-250, 50% methanol and 10% glacial acetic acid). Twenty min after staining with Coomassie blue, the membrane was washed in destaining solution (40% methanol and 10% glacial acetic acid) three times and rinsed with water until the background was fully destained.

### Infection of thioglycollate-elicited peritoneal macrophages

*Stat1*^*-/-*^ mice were injected intraperitoneally with 1 ml of 3% thioglycollate and peritoneal cells were collected on day 4 after injection as previous described [20]. Thioglycollate-elicited peritoneal cells were cultured in RPMI medium containing penicillin/streptomycin, sodium pyruvate, L-glutamine, MEM non-essential amino acids, 2-mercaptoethanol and supplemented with 10% heat-inactivated fetal bovine serum. Cells were allowed to adhere to the wells of 96-well plate overnight. The cell monolayers were rinsed with PBS and infected with culture-DENV2 or msq-DENV2-P1 at MOI of 20. Cells were then collected at 6, 24 and 48 h after infection.

### Semi-quantitative Reverse transcription (RT) PCR analysis

Total RNA was extracted from thioglycollate-elicited peritoneal macrophages that were infected with or without DENV2. Extracted RNA was reversely transcribed into cDNA. The primers for *Il6*, *Il10* and *Tnf* are listed in S1 Table. The ABI 7900HT Fast Real-Time PCR System was used for semi-quantitative PCR analysis (Applied Biosystems instruments, USA). Each reaction contained 20 μl mixture, including 4.2 μl of ddH_2_O, 10 μl of SensiFAST SYBR Hi-ROX Mix, 0.4 μl of forward primer, 0.4 μl of reverse primer and 5 μl of cDNA. The program used for semi-quantitative PCR analysis was 95°C for 3 min, followed by 95°C for 5 sec and 60°C for 30 sec in total of 40 cycles. The expression levels of *Il6*, *Il10* and *Tnf* transcripts were normalized against mouse *Gapdh*.

### Statistical analysis

Differences between two groups were analyzed with Wilcoxon-Mann-Whitney test and that for multiple comparisons were analyzed by one-way ANOVA with multiple comparisons test. The number of biological repeats and significant differences were shown in the corresponding figure legends. Values were presented as mean ± standard deviation (SD). All statistics analyses were calculated by GraphPad Prism 8 software.

## Results

### DENV2 released in infectious mosquito saliva has higher hemorrhage induction potential than DENV2 grown in cell culture

**Fig 1:**
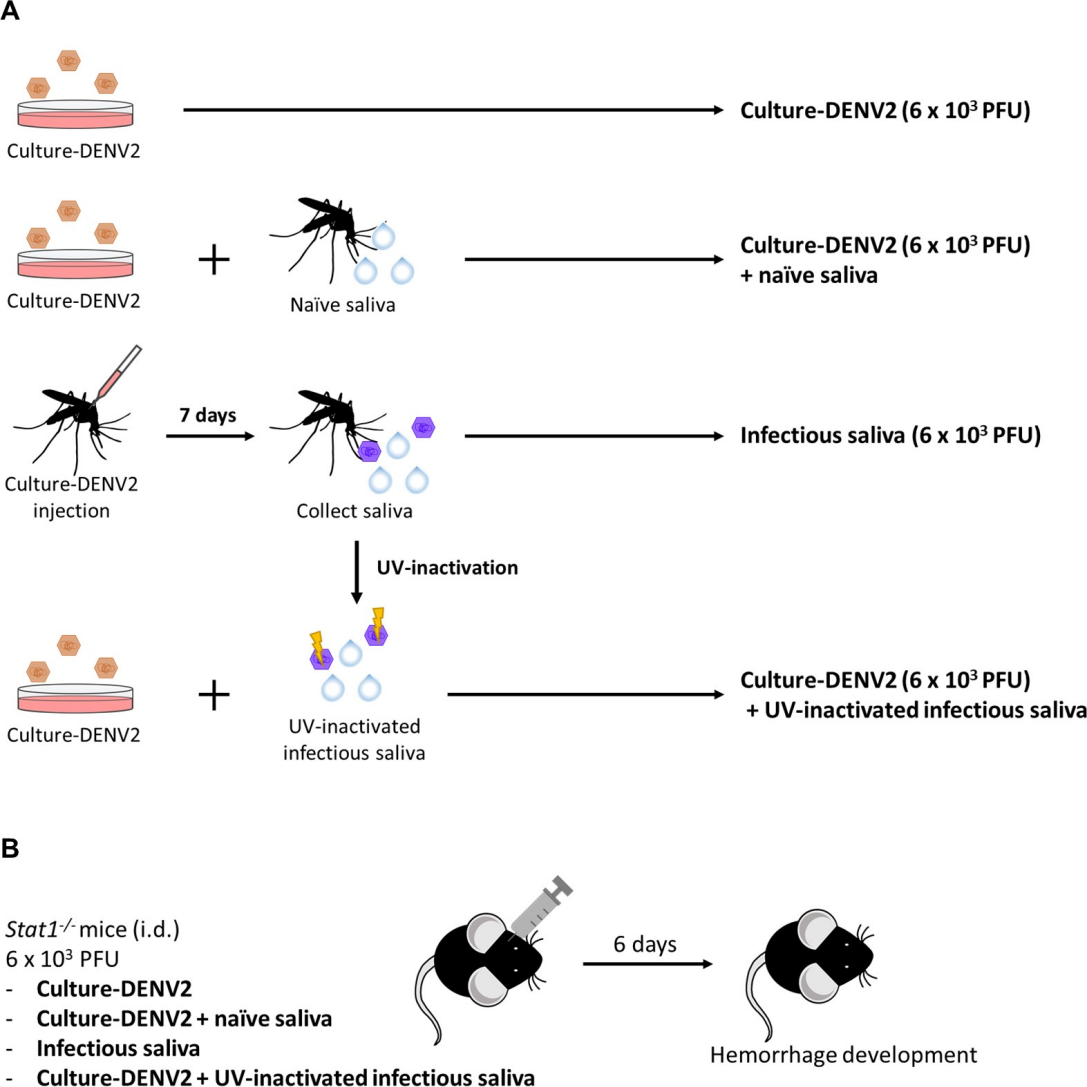
Scheme depicting study design. The experiment included four groups. (A, B) Inocula prepared in (A) were given to 4 groups of mice as in (B). (A) DENV2-16681 was propagated in C6/36 insect cell line and culture supernatants were collected and virus titer was determined by plague assay (culture-DENV2). Culture-DENV2 was mixed with saliva collected from naïve *Aedes aegypti* (culture-DENV + naïve saliva). *A*. *aegypti* was intrathoracically inoculated with 69 nl of DENV-2 16681 (6.9 × 10^2^ PFU in 69 nl) and saliva was collected on day 7 after infection (infectious saliva). Culture-DENV2 was mixed with infectious saliva that was exposed to ultraviolet (UV) light (culture-DENV2 + UV-inactivated infectious saliva). The titer of DENV2 from either cell culture or infectious mosquitoes was determined by plaque assay and adjusted to 6 × 10^3^ PFU in 400 μl. (B) Four groups of *Stat1*^*-/-*^ mice were injected intradermally at 4 different sites on the upper back with inoculum prepared in (A) in a total volume of 400 μl. Hemorrhage development was observed on day 6 after inoculation.

**Fig 2:**
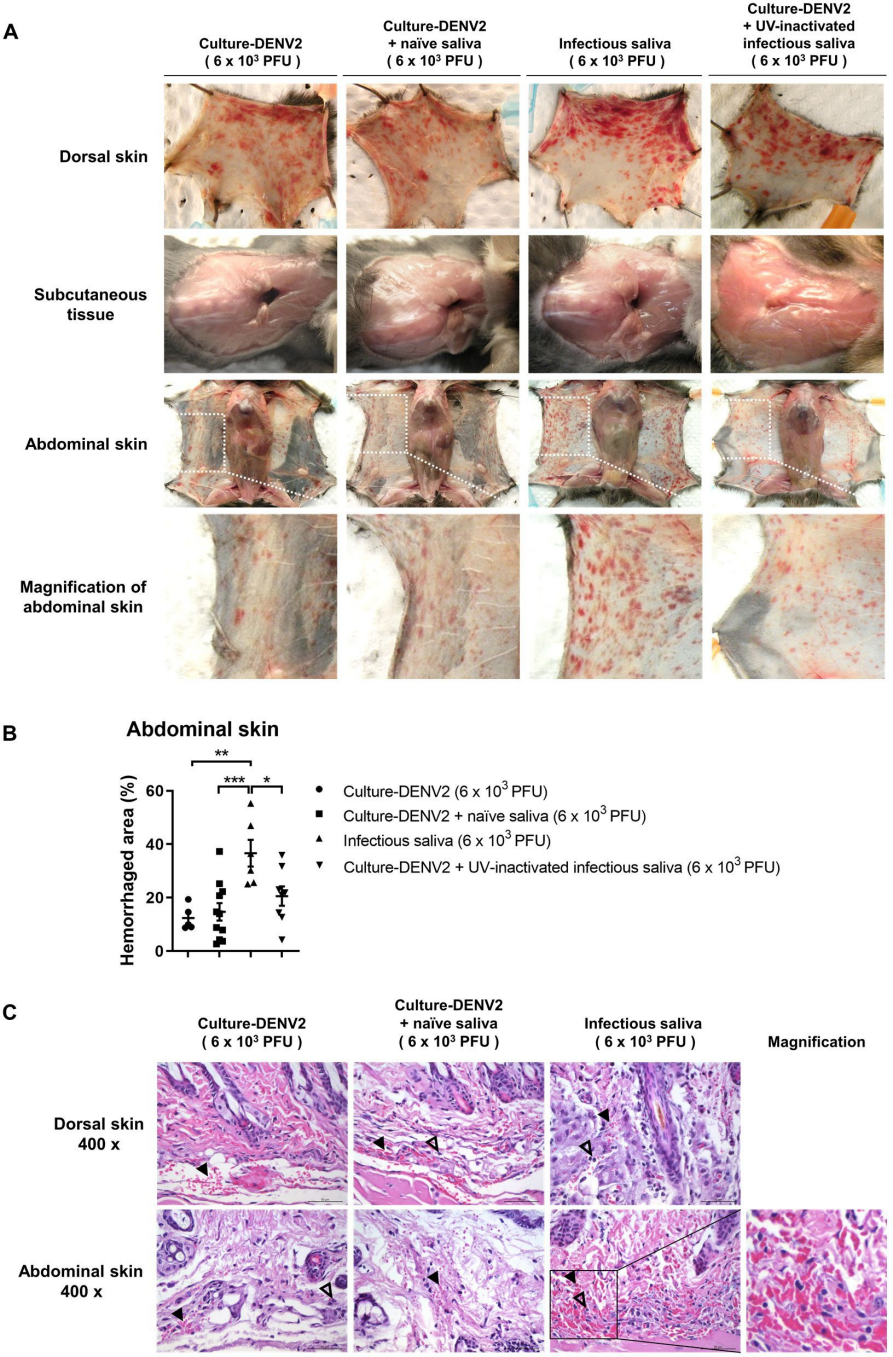
DENV2 from infectious mosquito saliva is more efficient in inducing hemorrhage than DENV2 grown in mosquito cell culture. Four groups of mice were inoculated with DENV2 following the protocol as described in Fig 1. (A) Mouse dorsal skin, subcutaneous tissues, and abdominal skin were dissected on day 6 after inoculation. The abdominal skin was magnified. (B) Hemorrhaged areas in the abdominal skin were quantified by ImageJ. Percentage of hemorrhaged area was obtained by dividing the sum of the hemorrhaged area in the selected region by the total selected area of the region. Approximately 60% of the total hemorrhaged areas of the abdominal skin was measured. Data presented in bar graphs are the mean ± SD obtained from four independent experiments (mice injected with culture-DENV2, n = 5; mice injected with culture-DENV2 + naïve saliva, n = 11; mice injected with infectious saliva, n = 6; mice injected with culture-DENV2 + UV-inactivated infectious saliva, n = 8). *p < 0.05, **p < 0.01, ***p < 0.005, as analyzed by one-way ANOVA with Dunn’s multiple comparisons test, comparing mice injected with infectious saliva with those injected with culture-DENV2, culture-DENV2 mixed with naïve saliva and culture-DENV2 mixed with UV-inactivated infectious saliva. (C) Dorsal and abdominal skins were fixed in formaldehyde, cut into 4 μm sections and stained with hematoxylin-eosin (H&E) stain. Filled triangles point to extravascular RBC and empty triangles to mononuclear cells. Area in the square was magnified showing red blood cell extravasation and leukocyte infiltration.

We employed dengue hemorrhage mouse model to investigate whether infection of mosquitoes causes any change in DENV2 that affects the ability of the virus to induce hemorrhage development in the mammalian host. As outlined in Fig 1A, saliva was collected from naïve (naïve saliva) and DENV2-infected mosquitoes (infectious saliva) [15]. *Stat1*^*-/-*^ mice which had been determined for their high susceptibility to low dose of DENV (S1 Fig) were divided into four groups: mice that were intradermally injected with (I) DENV2 grown in cell culture (culture-DENV2), (II) culture-DENV2 pre-mixed with saliva from naïve mosquitoes, (III) saliva from DENV2-infected mosquitoes (infectious saliva containing DENV2), and (IV) culture-DENV2 premixed with UV-treated infectious saliva. Each mouse was injected with 6 × 10^3^ PFU of DENV2 regardless whether the virus was obtained from cell culture or from infected mosquitoes (Fig 1B). Fig 2A shows that hemorrhage developed in dorsal and abdominal skins but not subcutaneous tissues in all four groups of mice on day 6 after infection. Mice that were inoculated with culture-DENV2 alone, culture-DENV2 pre-mixed with naïve mosquito saliva and culture-DENV premixed with UV-inactivated infectious saliva had similar level of severity in hemorrhage manifestation in both dorsal and abdominal skins. Interestingly, mice given infectious saliva developed more severe hemorrhage with greater areas of involvement in the abdominal skin than other groups of mice (Fig 2A and 2B). Consistent with our previous finding, hematoxylin-eosin stain revealed that there was red blood cell extravasation and leukocyte infiltration in the hemorrhaged skin (Fig 2C) [15]. While mice given infectious saliva had more severe red blood cell extravasation and greater number of leukocytes infiltrating the abdominal skin compared to mice given culture-DENV2 alone and culture-DENV2 premixed with naïve saliva, the levels of red blood cell extravasation and leukocyte infiltration were similar in dorsal skins from all other three groups of mice (Fig 2C). In line with severe hemorrhage development, 2 out of 6 mice injected infectious saliva developed viremia while none of the mice in the other groups did (S2 Fig). These results suggest that DENV2 in infectious mosquito saliva (msq-DENV2) has higher hemorrhage induction potential than virus obtained from cell culture and that mosquito saliva, whether it is from naïve or infected mosquitoes, does not potentiate hemorrhage induction ability of culture-DENV2 in *Stat1*^*-/-*^ mice.

### One cell-culture passage of DENV2 obtained from infectious saliva does not reduce its ability to induce hemorrhage in mice

**Fig 3:**
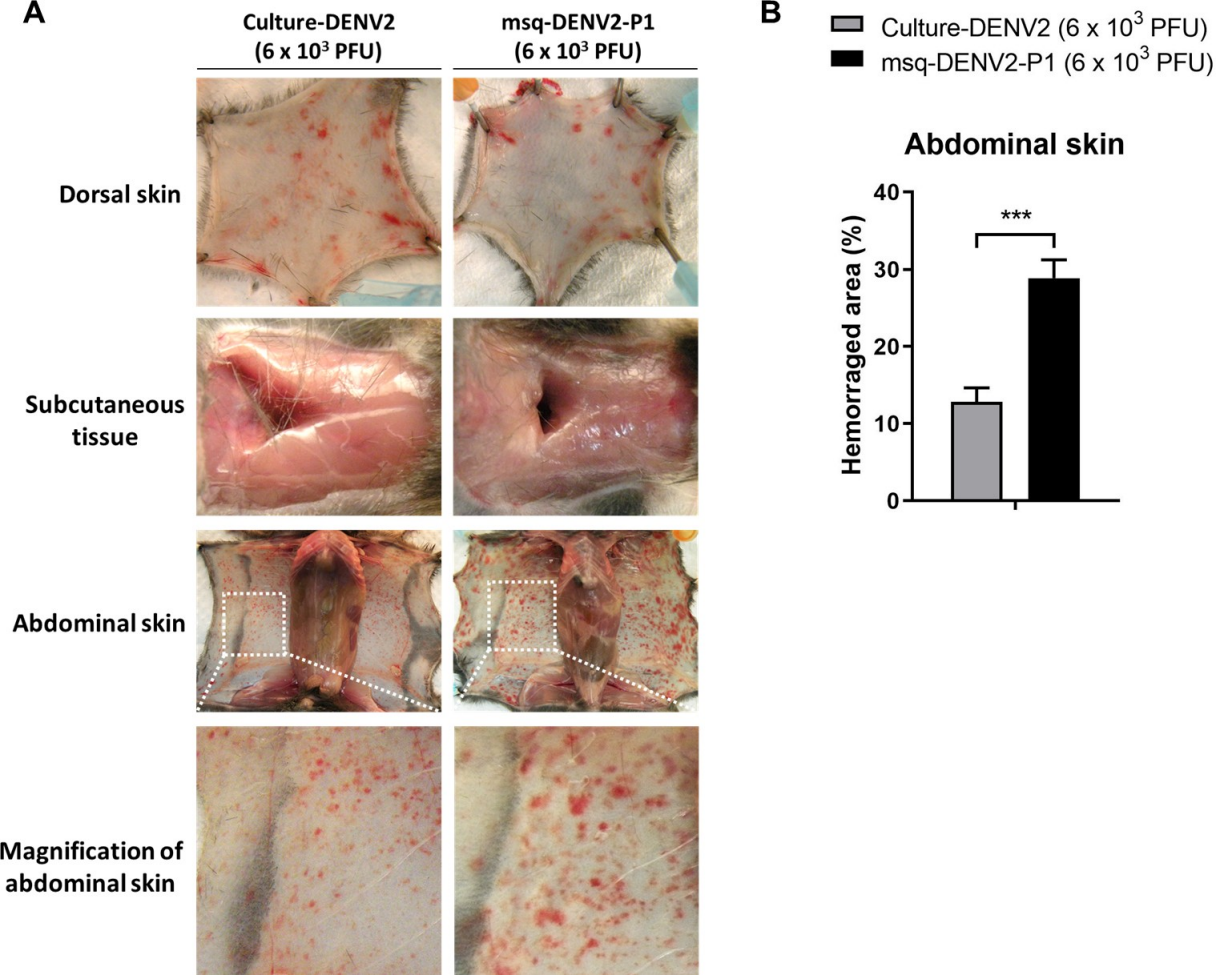
DENV2 isolated from infectious mosquito saliva retains hemorrhage-induction potential after one passage in mosquito cell line. Msq-DENV2 was propagated in C6/36 cell line. Culture supernatants containing the virus (msq-DENV-P1) was titered. *Stat1*^*-/-*^ mice were injected intradermally with 6 × 10^3^ PFU in 400 μl as described above. (A) Dorsal skin, subcutaneous tissues, and abdominal skin were observed on day 6 after inoculation. Representative photos of one mouse from each group is shown. Area in the square in abdominal skin was magnified. (B) Hemorrhaged areas were quantified by ImageJ. Percentage of hemorrhaged area was calculated as in Fig 2. Approximately 60% of the total hemorrhaged areas of the abdominal skin was measured. Data presented in the bar graphs are the mean ± SD obtained from 3 mice. ***p < 0.005, as analyzed by Wilcoxon–Mann–Whitney test, comparing mice inoculated with culture-DENV2 and msq-DENV2-P1.

We further explored the involvement of mosquito saliva in potentiating DENV2 hemorrhage-inducing potential. Infectious mosquito saliva (containing msq-DENV2) was placed on C6/36 cell monolayers and the supernatant was harvested 7 days later. Virus titer in the supernatants was determined by plaque assay. Virus thus obtained after one cell culture passage (msq-DENV2-P1) was again compared to culture-DENV2 for their potential to induce hemorrhage in *Stat1*^*-/-*^ mice. Mice were injected intradermally with culture-DENV2 or msq-DENV2-P1 at the same titer (6 × 10^3^ PFU). Hemorrhage development in both the dorsal and abdominal skins was observed on day 6 after inoculation (Fig 3A). Notably, mice receiving msq-DENV2-P1 developed more severe hemorrhage in the abdominal skin than those given culture-DENV2 (Fig 3A and 3B). These results together with those from Fig 2 indicate that DENV2 infecting mosquitoes acquires greater potential that is independent of the effect of mosquito saliva to induce hemorrhage in mice. Moreover, msq-DENV2 retains its hemorrhage potentiation ability after one passage in mosquito cell line.

### Infection of mosquito changes DENV2 virion size and growth characteristics

**Fig 4:**
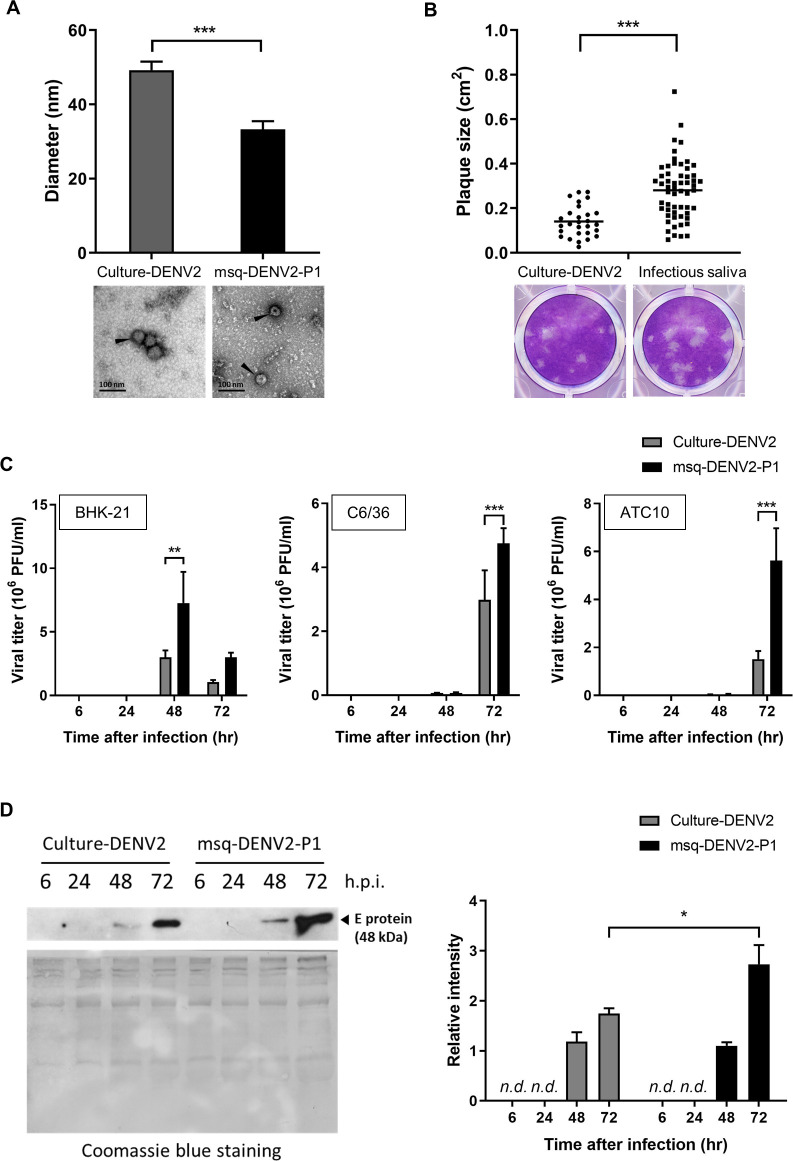
DENV2 isolated from infectious mosquito saliva are smaller in size, forms larger plaques and grows faster than DENV2 grown in mosquito cell culture. (A) Dengue virions from culture-DENV2 and msq-DENV2-P1 were observed by transmission electron microscopy. The diameter of dengue virions was measured by ImageJ. Representative images are shown below the bar graph and S2 Fig. n = 14 for each group. *** *p* < 0.005, by comparing culture-DENV2 with msq-DENV2-P1, was analyzed by Wilcoxon–Mann–Whitney test. Arrowheads point to virions. (B) Culture-DENV2 and infectious saliva were added onto BHK-21 cell monolayers separately. Five days later, plaque size was measured by ImageJ. Data presented in the bar graph are the mean ± SD obtained from 3 independent experiments and analyzed by Wilcoxon–Mann–Whitney test. Representative images are shown below the graph and in S3 Fig. (C) BHK-21 cells were infected with culture-DENV2 or msq-DENV2-P1 at a MOI of 0.01. C6/36 and ATC10 cells were infected with culture-DENV2 or msq-DENV2-P1 at a MOI of 0.1. Viral titers were determined by plaque assay. One-way ANOVA with Dunn’s multiple comparisons test was used to compare the differences in viral titers between culture-DENV2 and msq-DENV2-P1 with data from multiple time points. (D) ATC10 culture supernatants collected at different time points were analyzed for E protein expression by Western blot (upper gel). Coomassie blue staining shows the total protein loaded onto each lane (bottom gel). The relative intensity of E protein in the supernatants is presented in the bar graphs as mean ± SD pooled from 4 independent experiments. *n*.*d*. = non-detected, *p < 0.05, **p < 0.01, ***p < 0.005 comparing culture-DENV2 with msq-DENV2-P1. One-way ANOVA with Dunn’s multiple comparisons test was used to compare the differences in E protein intensity between culture-DENV2 and msq-DENV2-P1 with data from multiple time points.

We compared the morphology of msq-DENV2-P1 virions to that of culture-DENV2. DENVs were 10-fold concentrated and subjected to TEM analysis. As shown in Figs 4A and S4, the diameter of msq-DENV2-P1 viral particles was significantly smaller than culture-DENV2, and there was no obvious difference in the shape of the virions between the two. Moreover, the plaque size of msq-DENV2 was significantly larger (0.28 ± 0.13 cm^2^) than that of culture-DENV2 (0.14 ± 0.07 cm^2^) (Figs 4B and S5). Culture-DENV2 and msq-DENV2-P1 were allowed to infect mammalian cell line BHK-21, and mosquito cell lines C6/36 and ATC10. Plaque assay results in Fig 4C show that msq-DENV2-P1 grew to significantly higher titers than culture-DENV2 in all three cell lines we studied. Western blot analysis of culture supernatants showed that msq-DENV2-P1-infected ATC10 released higher levels of E protein than cells infected by culture-DENV2 at 72 h after infection (Fig 4D), confirming that msq-DENV2-P1 replicates more efficiently than culture-DENV2 in cell culture [19]. These results together indicate that infecting mosquitoes alter the growth characteristics of DENV2. The change is shown in its particle size, plaque size and growth which effects greater hemorrhage induction potential.

### Msq-DNEV2 is more efficient in stimulating macrophage TNF response than culture-DENV2

**Fig 5:**
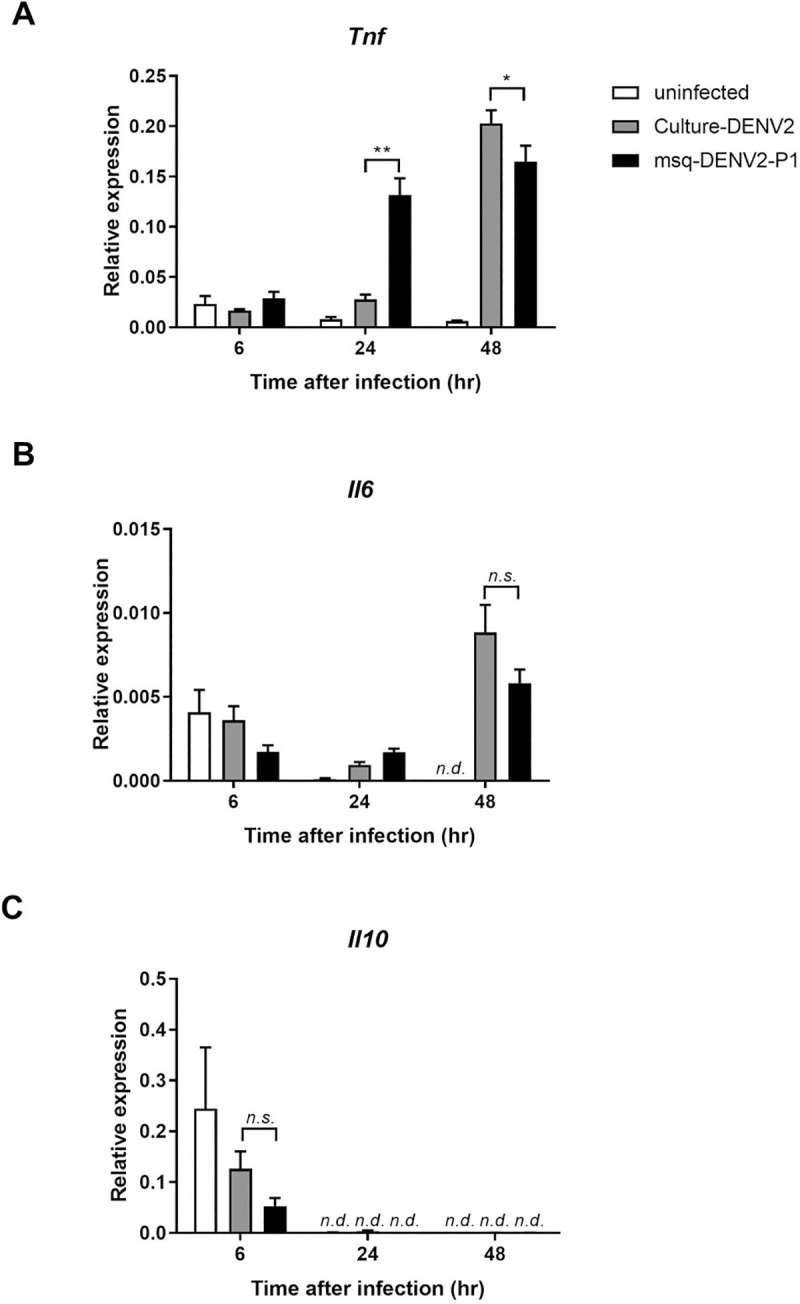
Comparing msq-DENV2 and culture-DENV2 for their ability to induce cytokines in mouse macrophages. Peritoneal macrophages were obtained from *Stat1*^*-/-*^ mice on day 4 after intraperitoneal injection of thioglycollate (Thio-pMac). Thio-pMac were stimulated with culture-DENV2 or msq-DENV2-P1 at a MOI of 20 for 6, 24 or 48 h. RNA was extracted, reverse-transcribed to cDNA for real-time PCR quantitation. mRNA of each cytokine was normalized against mouse *Gapdh*. n = 4 for each group for each time point. *n*.*s*. = non-significant, ***p < 0.005, as analyzed by one-way ANOVA with Dunn’s multiple comparisons test comparing cytokine levels induced by culture-DENV2 and msq-DENV2-P1 from different time points.

Our previous study showed that TNF produced by infiltrating macrophages is critical to hemorrhage development in DENV mouse model [21]. We compared msq-DENV2-P1 to culture-DENV2 for their ability to induce macrophage *Tnf* production. Thioglycollate-elicited macrophages from *Stat1*^*-/-*^ mice were stimulated with DENV2 from the two different sources at MOI of 20. RT real-time PCR results showed that msq-DENV2-P1 induced almost 4-fold higher *Tnf* than culture-DENV did at as early as 24 h after stimulation when culture-DENV-stimulated *Tnf* production still remained marginal, showing that msq-DENV2-P1 is more efficient than culture-DENV2 in triggering *Tnf* production (Fig 5A). There was no significant difference between msq-DENV2-P1 and culture-DENV2 in their stimulations of *Il6* and *Il10* responses (Fig 5B and 5C). These data demonstrate a correlation between the efficiency of msq-DENV2 in inducing *Tnf* production and its ability to potentiate hemorrhage development. We speculate that infecting mosquito alters DENV that it becomes more efficient in triggering *Tnf* thus has greater potential to induce hemorrhage in mice.

## Discussion

In this study, we discovered a vital role of mosquito vectors in the infection dynamics of DENV. *A*. *aegypti* mosquito plays an active role in altering the characteristics of the infecting DENV as well as its potential to cause hemorrhage in mice. These observations were made possible by our unique approach of collecting saliva and virus from large numbers of infected mosquitoes, thereby enabling us to characterize the virus and consequently its effect on disease manifestation in the mammalian host.

To study the effect of mosquito salivary proteins on the response of mammalian host to infection, investigators conducted their studies by co-injecting mosquito salivary gland extracts (SGE) [22] or purified mosquito saliva from naïve mosquito [7, 19, 23] together with virus or by allowing mosquitoes to bite experimental animals before injecting virus [7,8,23,24]. SGE contains not only saliva but also secreted and non-secreted salivary proteins as well as disrupted mosquito cells. These additional mosquito elements that may or may not be delivered during mosquito probing, modulate host immune response to the virus [8,9,24,25]. Amongst these approaches, injecting saliva to the host is closest to probing if one disregards the difference in mechanical damage caused by needle injection and probing. Conventionally, mosquito saliva is collected by allowing individual mosquitoes to salivate into a capillary tube [25]. The small volume of saliva collected by this method makes it difficult to quantify and characterize the virus contained in the saliva. The method we collect saliva from mosquitoes in this study has recently been published [15]. DENV-infected mosquitoes are fed on a parafilm-wrapped artificial feeder and allowed to salivate into the feed. Saliva from as many as 200 mosquitoes can be simultaneously collected, enough quantity to determine viral titer, plaque size and growth characteristics. Saliva collected from infected mosquitoes by this method contained approximately 1 × 10^5^ PFU/ml after 4-5-fold concentration, a titer high enough to conduct in vivo experiments in the mammalian host. This approach is useful for studies involving interactions between virus and the mosquito vector as well as its consequential effect on disease progression in the host.

Human is a natural host of DENV. DENV NS2B3 protease inhibits type I IFN signaling, enabling the virus to efficiently infect the human host [26,27]. While the proteolytic activity of NS2B3 protease degrades the key adaptor protein of type I interferon pathway, human mediator of IRF3 activation (MITA), it is not able to degrade its mouse counterpart MPYS [27]. This phenomenon limits the usefulness of immunocompetent mice to study dengue. We established a dengue hemorrhage mouse model in immunocompetent mice that requires intradermal inoculation of high titers (10^7^ ~ 10^9^ PFU) of DENV for hemorrhage manifestation [10], a titer that is not achievable by isolating virus from infected mosquito saliva. Immunodeficient mice including AG129 mice, *Ifnar*^*-/-*^ mice, IRF3^-/-^7^-/-^ and *Stat1*^*-/-*^ have been demonstrated to be susceptible to DENV infection [8,11–14]. JAK/STAT signaling is critical for the production of antiviral proteins and pro-inflammatory cytokines [28]. Mice that lack the *Stat1* gene are defective in their ability to respond to both type I and II IFNs and are highly susceptible to viral and intracellular bacterial infections [14,29–32]. Our study showed that *Stat1*^-/-^ mice are responsive to gradient titers of DENV2 and the severity of hemorrhage development correlates with the titers of the viral inocula (S1 Fig) delivered through the intradermal route. These results support the notion that *Stat1*^*-/-*^ mice and possibly other mice deficient in type I IFN signaling can be applied to investigate the subtle differences in pathogenicity of different DENV isolates, especially in hemorrhage manifestations. Hemorrhaged areas as quantified by ImageJ software can be used as an indicator for DENV pathogenicity.

To demonstrate the consequential effect of DENV collected in infectious saliva on mammalian host, it is important to delineate the effect of infectious mosquito saliva and that of the virus. Saliva was collected from infected mosquitoes and UV-irradiated to inactivate the virus. Naïve saliva and UV-irradiated infectious saliva were separately mixed with culture-DENV2 before injecting *Stat1*^*-/-*^ mice. No difference was observed in the severity of hemorrhage manifestations between these two groups and that of control mice receiving culture-DENV. These results suggest that mosquito saliva, either from naïve or from infected mosquitoes, does not affect DENV2-induced hemorrhage in the mammalian host (Fig 2). In addition, infectious mosquito saliva was diluted and plated on C6/36 cells at MOI of 0.01 to allow virus propagation. Msq-DENV2-P1 in the culture supernatant, like its parental msq-DENV2, has higher potency than culture-DENV2 in inducing hemorrhage development (Fig 3). By these two experiments, we rule out the effect of mosquito saliva and demonstrate that DENV2 became more potent after infecting mosquitoes in inducing hemorrhage in mice.

RNA viruses are known to have high mutation rates due to the error-prone nature of viral RNA-dependent RNA polymerase [33]. The viral population is consisted of high numbers of variant genomes called quasispecies. Genetic mutation that changes the composition of quasispecies impacts viral replication, plaque morphology and the pathogenesis of the disease it causes [34–36]. We observed that after infecting mosquitoes msq-DENV2 displays larger plague size compared to the original inoculum (culture-DENV2). After one cell culture passage in C6/36 cells, msq-DENV2-P1 collected from culture supernatants retains the ability to induce severe hemorrhage as its parental msq-DENV2 and exhibits smaller virion size, higher growth rate in both mammalian and mosquito cell lines as well as greater efficiency than culture-DENV2 in inducing *Tnf* production in mouse macrophages. These observations suggest that spontaneous mutation may occur to DENV2 in the mosquito vector thus impacts the composition of quasispecies, resulting in alteration of the characteristics of the virus. Although it remains to be determined whether there are mutation hotspots in the viral genome and what the hotspots are, to our knowledge this study is the first to show that the mosquito vector provides an environment that allows DENV2 to go through genetic diversification for greater virulence. As DENV has four serotypes and *A*. *albopictus* is also a vector that transmits the virus, further experiments is required to determine whether serotypes 1, 3, and 4 also undergo changes in the mosquito vector, be it *A*. *aegypti* or *A*. *albopictus*. We reported previously that silencing the expression of salivary protein AsSG34 in *A*. *agypti* reduces DENV2 replication in the salivary gland and transmission of the virus to *Stat1*^*-/-*^ mice [19]. Buchman *et al*. created *A*. *agypti* mosquitoes carrying a gene encoding an engineered single-chain variable fragment derived from a broadly neutralizing DENV human monoclonal antibody [37]. These mosquitoes have reduced vector competence and limited ability to acquire and transmit pathogens. In light of our finding that *A*. *aegypti* vector allows DENV2 to become more virulent, it is our speculation that disrupting viral growth in and blocking transmission of the virus by mosquitoes through molecular biological means may be a promising strategy to control the spread of dengue.

By devising a unique method of collecting saliva from a large number of mosquitoes and employing inoculum size-sensitive *Stat1*^*-/-*^ mice as a model for dengue hemorrhage, we addressed whether the mosquito vector changes DENV2 and affects its virulence in causing disease in the mammalian host. Our results suggest that the mosquito vector facilitates changes in DENV2, possibly by changing the composition of the quasispecies. Compared to culture-DENV2, msq-DENV2 is smaller in size, forms larger plaques and grows at a higher rate in both mosquito and mammalian cell cultures. Remarkably, accompanying its efficiency in inducing *Tnf* in mouse macrophages, msq-DENV2 induces more severe hemorrhage in mice. Our work sheds light on the importance of the mosquito vector in DENV genetic change during transmission cycle.

## Supporting information

S1 Fig Intradermal inoculation of DENV2 at as low as 4 x 10^3^ PFU induces hemorrhage in *Stat1^-/-^* mice. *Stat1*^*-/-*^ mice were injected intradermally with gradient doses of DENV2 at four different sites on the upper back. Hemorrhage development on dorsal skin and abdominal skin was observed on day 8 after inoculation.
(TIF)

S2 Fig *Stat1^-/-^* mice develop viremia after intradermal inoculation with infectious saliva. *Stat1*^*-/-*^ mice were injected intradermally with culture-DENV2, culture-DENV2 pre-mixed with naïve saliva, saliva obtained from DENV-infected mosquitoes (infectious saliva) or culture-DENV2 pre-mixed with infectious saliva that was exposed to ultraviolet (UV) light (culture-DENV2 + UV-inactivated infectious saliva). Mouse serum was collected on day 6 after infection and the viral titer was determined by plaque assay in BHK-21 cell line.
(TIF)

S3 Fig Measurement of hemorrhaged areas. Hemorrhaged areas (yellow outline) were measured by ImageJ software. Percent hemorrhaged area was calculated by dividing the hemorrhaged area in the selected region by the total area of the selected region.
(TIF)

S4 Fig msq-DENV-2-P1 virion is smaller in size than culture-DENV2. Dengue virions from (A) culture-DENV2 and (B) msq-DENV2-P1 were observed under transmission electron microscope. Particle size was measured by ImageJ. Arrowheads point to virions.
(TIF)

S5 Fig DENV2 isolated from infectious mosquito saliva forms larger plaques than DENV2 grown in mosquito cell culture. (A) Culture-DENV2 and infectious saliva were added to BHK-21 cell monolayers separately. Five days later, the cells were fixed by 4% paraformaldehyde and stained with 1% crystal violet. (B) The area of the plaques (yellow outline) was quantified by ImageJ. Plaque size = (pixel number of the plaque/pixel number of the whole well) × the total area of whole well (1.9 cm^2^).
(TIF)

S1 Table The primers used for semi-quantitative PCR analysis. (PDF)
