# Supplementary figures and images for: Infecting mosquitoes alters DENV-2 characteristics and enhances hemorrhage-induction potential in Stat1-/- mice
Source: PLoS Negl Trop Dis. 2021 Aug 27;15(8):e0009728. doi: 10.1371/journal.pntd.0009728 (PMC8428656; doi:10.1371/journal.pntd.0009728)

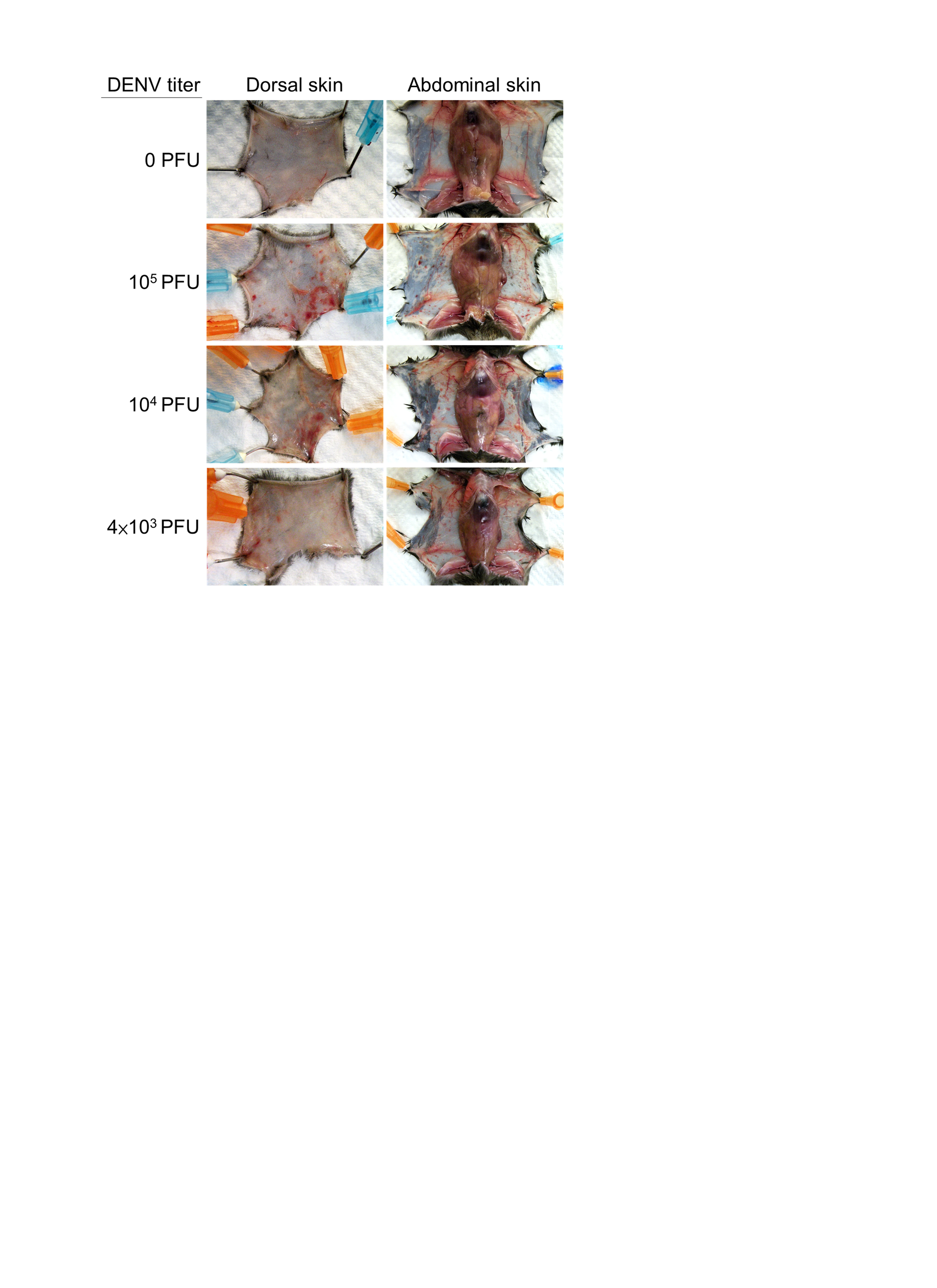

Supplement: S1 Fig — Stat1-/- mice were injected intradermally with gradient doses of DENV2 at four different sites on the upper back. Hemorrhage development on dorsal skin and abdominal skin was observed on day 8 after inoculation. (TIF) [file pntd.0009728.s001.tif]

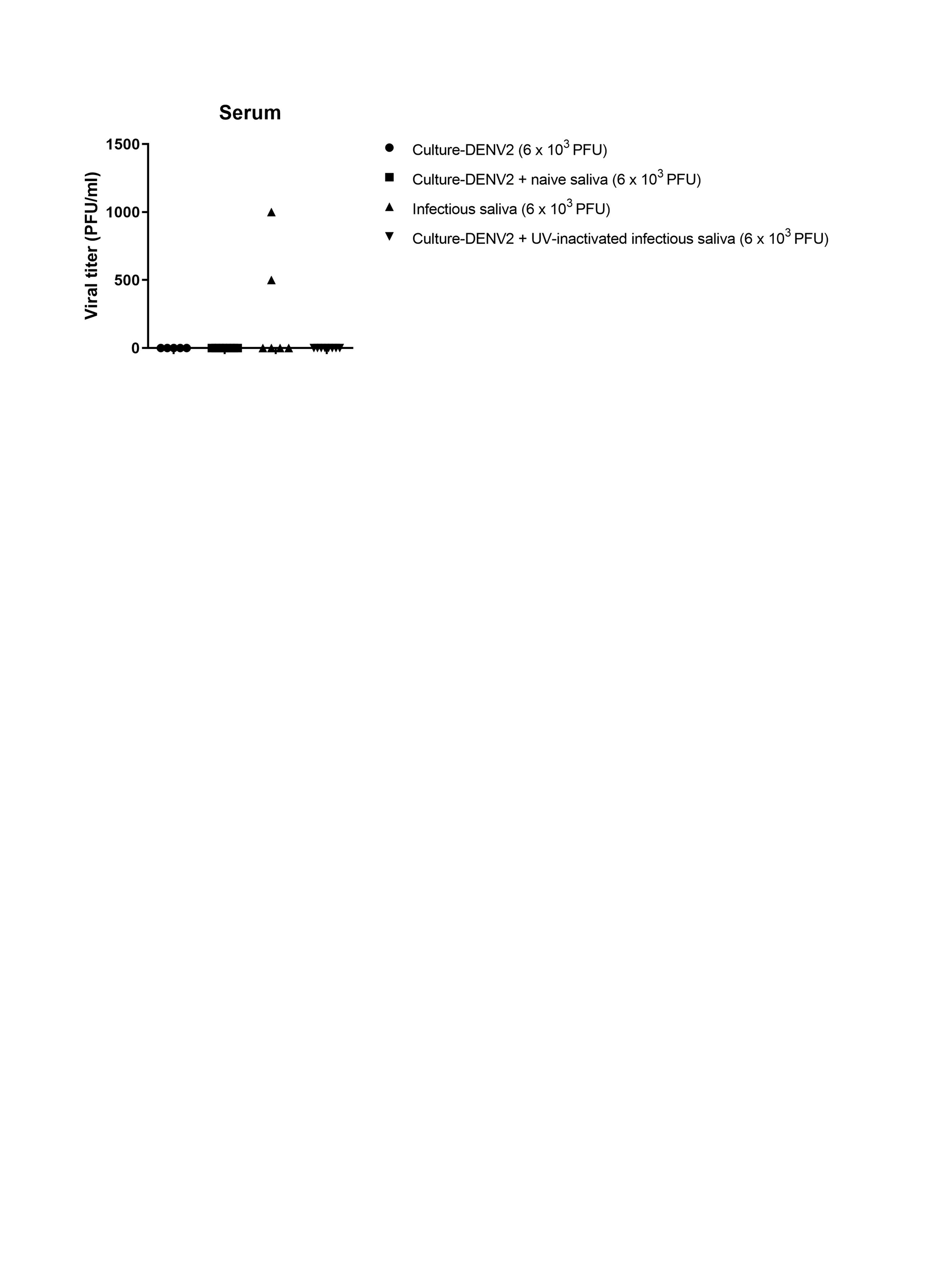

Supplement: S2 Fig — Stat1-/- mice were injected intradermally with culture-DENV2, culture-DENV2 pre-mixed with naïve saliva, saliva obtained from DENV-infected mosquitoes (infectious saliva) or culture-DENV2 pre-mixed with infectious saliva that was exposed to ultraviolet (UV) light (culture-DENV2 + UV-inactivated infectious saliva). Mouse serum was collected on day 6 after infection and the viral titer was determined by plaque assay in BHK-21 cell line. (TIF) [file pntd.0009728.s002.tif]

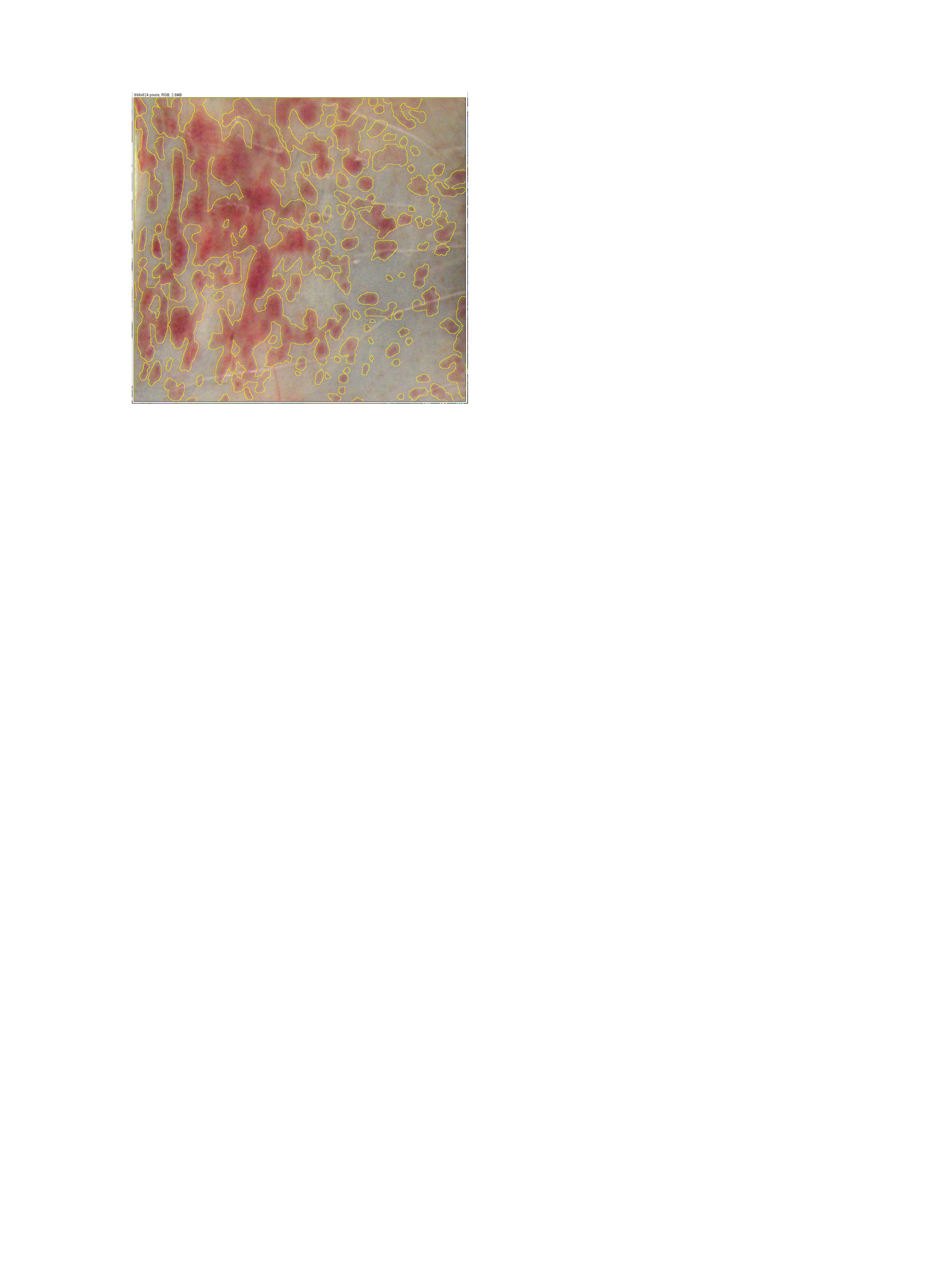

Supplement: S3 Fig — Hemorrhaged areas (yellow outline) were measured by ImageJ software. Percent hemorrhaged area was calculated by dividing the hemorrhaged area in the selected region by the total area of the selected region. (TIF) [file pntd.0009728.s003.tif]

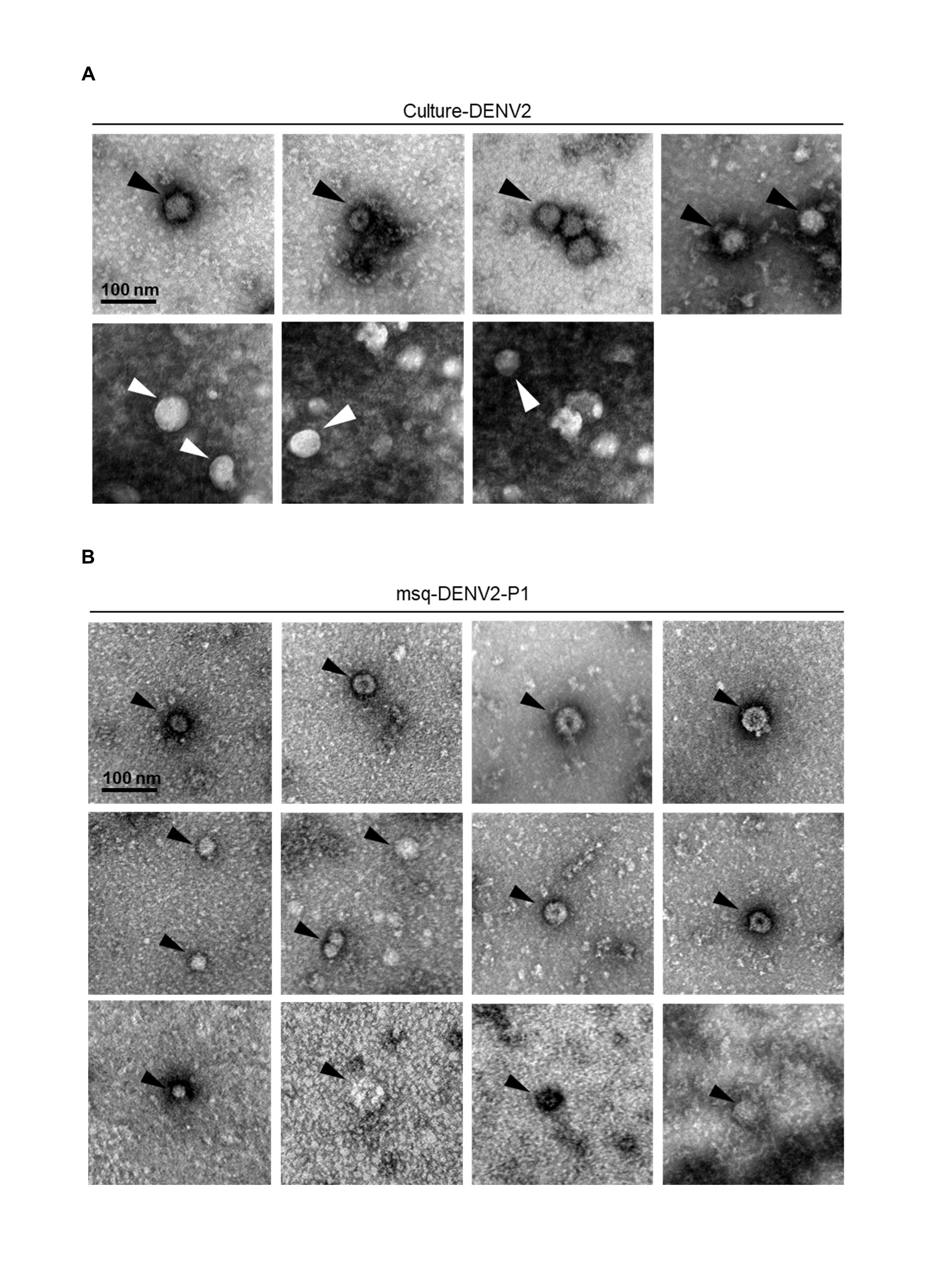

Supplement: S4 Fig — Dengue virions from (A) culture-DENV2 and (B) msq-DENV2-P1 were observed under transmission electron microscope. Particle size was measured by ImageJ. Arrowheads point to virions. (TIF) [file pntd.0009728.s004.tif]

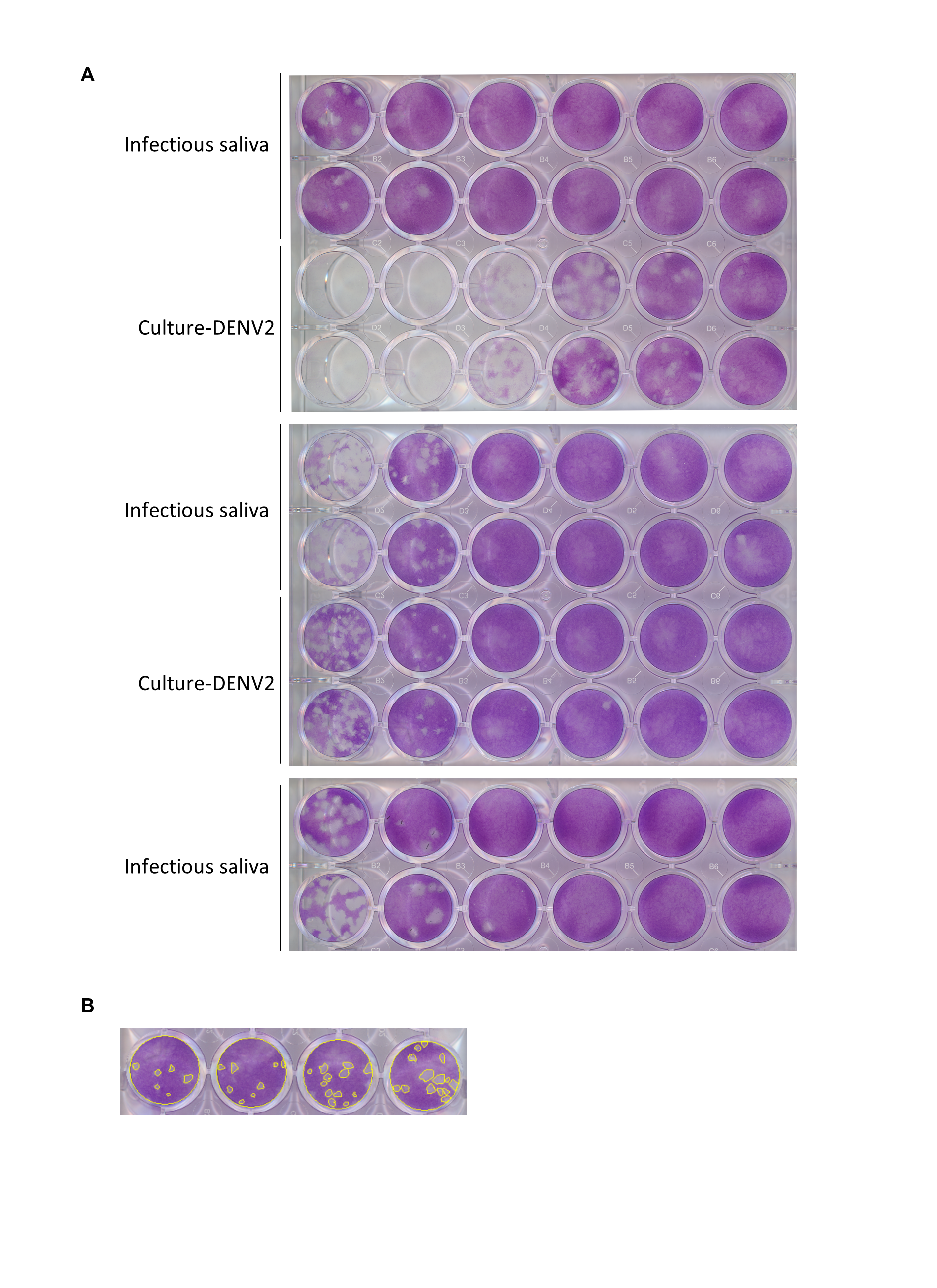

Supplement: S5 Fig — (A) Culture-DENV2 and infectious saliva were added to BHK-21 cell monolayers separately. Five days later, the cells were fixed by 4% paraformaldehyde and stained with 1% crystal violet. (B) The area of the plaques (yellow outline) was quantified by ImageJ. Plaque size = (pixel number of the plaque/pixel number of the whole well) × the total area of whole well (1.9 cm2). (TIF) [file pntd.0009728.s005.TIF]
