# Supplementary material for: Infecting mosquitoes alters DENV-2 characteristics and enhances hemorrhage-induction potential in Stat1-/- mice
Source: PLoS Negl Trop Dis. 2021 Aug 27;15(8):e0009728. doi: 10.1371/journal.pntd.0009728 (PMC8428656; doi:10.1371/journal.pntd.0009728)
Supplement: S1 Table — (PDF) [file pntd.0009728.s006.pdf]

S1 Table. The primers used for quantitative PCR analysis.

Target (mouse)	Primer	Sequence
Gapdh	Forward	5’- GGCAAATTCAACGGCACAGT-3’
	Reverse	5’- AGATGGTGATGGGCTTCC-3’
Tnf	Forward	5’- CATCTTCTCAAAATTCGAGTGACAA-3’
	Reverse	5’- TGGGAGTAGACAAGGTACAACCC-3’
Il6	Forward	5’-GAGGATACCACTCCCAACAGACC-3’
	Reverse	5’-AAGTGCATCATCGTTGTTCATACA-3’
Il10	Forward	5’-GCCTTCAGTATAAAAGGGGGACC-3’
	Reverse	5’-GTGGGTGCAGTTATTGTCTTCCCG-3’
